# Gene-gene interactions lead to higher risk for development of type 2 diabetes in a Chinese Han population: a prospective nested case-control study

**DOI:** 10.1186/s12944-018-0813-6

**Published:** 2018-07-28

**Authors:** Wen Zhou, Yuqian Li, Lulu Zhang, Yuanyuan Shi, Chongjian Wang, Dongdong Zhang, Xuejiao Liu, Zhenxing Mao, Linlin Li

**Affiliations:** 1 Department of Epidemiology and Health Statistics, College of Public Health, Zhengzhou University, 100 Kexue Avenue, Zhengzhou, 450001 Henan China; 2 Department of Clinical Pharmacology, School of Pharmaceutical Science, Zhengzhou University, 100 Kexue Avenue, Zhengzhou, 450001 Henan China

**Keywords:** Gene, Interaction, Haplotype, Type 2 diabetes mellitus, Nested case-control study

## Abstract

**Background:**

The purpose of this study was to evaluate the effect of single-nucleotide polymorphisms (SNPs) of the *GCKR* and *G6PC2* genes on risk for type 2 diabetes and the SNP-SNP and haplotype-based interactions between these genes.

**Methods:**

Subjects of this nested case-control study were selected from a prospective cohort residing in the rural area of Luoyang city in China. Cases (*n* = 538) were individually matched with controls. Six SNPs in the *GCKR* and *G6PC2* genes were selected and genotyped using an SNPscan™ kit. Stratified Cox proportional hazards regression models were used to generate odds ratios (ORs) and 95% confidence intervals (CI) for different genotype models for the risk of T2DM. Generalized multifactor dimensionality reduction (GMDR) was used to analyze the interactions between two genes with among six SNPs. The linkage disequilibrium (LD) analysis and the haplotype analysis were carried out by SHEsis online.

**Results:**

We found that the C allele of rs780094 was associated with increased risk for T2DM in Han Chinese population. However, the rs492594-C allele in *G6PC2* was associated with a decreased risk of T2DM. We also found a significant SNP-SNP interaction between rs2293572 and rs492594, and the CCCCGC and CGCCCA haplotypes significantly increased the risk of T2DM, however, the CCCCCA haplotype had lower susceptibility to T2DM.

**Conclusion:**

The results suggest that the *GCKR* and *G6PC2* genes may contribute to the risk of T2DM independently and/or in an interactive manner in the Han Chinese population.

**Electronic supplementary material:**

The online version of this article (10.1186/s12944-018-0813-6) contains supplementary material, which is available to authorized users.

## Background

Type 2 diabetes mellitus (T2DM) is a common but complex multifactorial chronic disease, accounting for > 95% of diabetes worldwide. [1] It is estimated that every year over 3.8 million people are dying of T2DM and its complications in worldwide. [2] The prevalence of T2DM presents a trend of sustainable growth over the past few decades. The International Diabetes Federation reported that the number of people with diabetes aged 20–79 years was expected to reach 642 million by 2040. [3] These observations show that T2DM is a major worldwide health problem.

With the rapidly rising prevalence of T2DM, a more systematic understanding of the natural history of the disease and its potential risk factors is urgently needed. Both genetic and environmental factors contribute to the occurrence and pathophysiology of T2DM. However, knowledge of the contribution of genetic factors to T2DM risk is still limited. The level of fasting plasma glucose (FPG) is the core factor that correlated with the risk of T2DM and various cardiovascular diseases. [4] There is strong evidence suggesting that hyperglycemia plays a key role in a concentration-dependent manner for both micro- and macro-vascular complications in diabetes. [5] Glucokinase regulatory protein (GCKR) and glucose-6-phosphatase catalytic subunit 2 (G6PC2) are both recognized glucose metabolism-related genes. Studies show that single-nucleotide polymorphisms (SNPs) of the *GCKR* and *G6PC2* genes are associated with FPG and T2DM incidence, although the conclusions are inconsistent in different regions. [6–9] Moreover, the SNPs associated with T2DM explain only a part of the heritability. Mechanisms such as gene-gene or gene-environment interactions may account for this missing heritability. [10, 11]. There have been reports of the relationship between the *GCKR* and *G6PC2* genes and FPG and T2DM, but the association between the interaction of these two genes and T2DM risk has not been reported.

Therefore, the aim of the present study was to detect associations between SNPs in *GCKR* and *G6PC2* and the risk for T2DM in a rural adult Chinese population, as well as to determine if gene-gene interactions modify the risk of T2DM incidence.

## Methods

### Study design and population

Subjects of this nested case-control study were selected from participants of a prospective cohort residing in the rural area of Luoyang city in China. The first phase of this cohort study was conducted from July to August of 2007 and July to August of 2008 on 20,194 subjects, and follow-up examinations were conducted from July to August 2013 to July and October 2014 to identify newly developed diseases. Details of this cohort study have been published elsewhere. [12, 13] T2DM was defined as FPG ≥7.0 mmol/L or the use of insulin or oral hypoglycemic agents, and/or a self-reported history of T2DM. [14] Subjects were > 25 and < 75 years old and of Northern Chinese ancestry. We excluded participants who had body mass index (BMI) < 18.5 Kg/m^2^; were pregnant, handicapped, or mentally illness; and had cancer or were unable or unwilling to participate. During the follow-up, we recruited 550 subjects (196 men and 354 female) who developed T2DM. Controls were matched in a 1:1 ratio to cases by age (within 2 years), sex, and village. Because the genotype of SNPs of part of objects have not been detected, 538 incident cases and 538 controls were selected in the end.

Data on demographic and anthropometric characteristics were collected by a standard interviewer-administered questionnaire. Anthropometric data included body weight, body height, BMI, WC, waist-height ratio and blood pressure. Blood pressure were measured by using an electronic sphygmomanometer.

### Biochemical measurements

All blood samples were collected from subjects in the morning after an overnight fast for measuring FPG, total cholesterol (TC), triglycerides (TG), and high-density lipoprotein cholesterol (HDL-C), as well as genotyping. FPG was measured using an oxidase enzymatic method, whereas TC, TG, and HDL-C were measured using an automatic biochemical analyzer (Hitachi, Tokyo, Japan). The concentration of low-density lipoprotein cholesterol (LDL-C) was calculated using the Friedewald formula. [15]

### Selection and genotyping of SNPs

The tag SNPs rs780094, rs2293572, rs12603206 and rs492594, rs16856187, rs13387347 for *GCKR* and *G6PC2* were selected for the Chinese population from the International HapMap project by use of the exact criteria of a minor allele frequency (MAF) > 0.01 and r^2^ ≥ 0.8. Genomic DNA was extracted from peripheral blood by use of a blood genome DNA purification kit (Yaneng BIO, Shenzhen, China). Genotype polymorphisms were identified by SNPscan™ kit (Genesky Biotechologies Inc., Shanghai, China). This kit was developed using patented SNP genotyping technology by Genesky Biotech Co., Ltd., involving the technology of double ligation and multiplex fluorescence polymerase chain reaction. The minimum call rate was 97.8%. To verify reproducibility, genotyping was reanalyzed based on 50 duplicates randomly selected from 1076 specimens and the concordance rate was more than 99%. All subjects underwent genotyping at baseline.

### Statistical analysis

Baseline data are summarized as the median with interquartile range for quantitative variables for data with non-normal distribution and number (percentage) for categorical variables. The Mann-Whitney Wilcoxon test was used to assess the significance of differences in quantitative variables and the chi-square test for categorical variables. The Hardy-Weinberg Equilibrium (HWE) was calculated by Pearson’s chi-square statistic test. Stratified Cox proportional hazards regression models were used to generate odds ratios (ORs) and 95% confidence intervals (CI) of different genotype models for the risk of T2DM adjusting for the baseline data including BMI, BMI-change, smoking and drinking status, and family history of diabetes. The interactions between the two genes for six SNPs were analyzed by statistical analysis and were performed using generalized multifactor dimensionality reduction (GMDR). SNP-SNP interaction analysis used unconditional logistic regression. Data were analyzed using SPSS v21.0 for Windows (SPSS Inc., Chicago, IL). The linkage disequilibrium (LD) analysis and the haplotype analysis were carried out by SHEsis online (http://analysis.bio-x.cn/myAnalysis.php). [16] Two-sided *p* <  0.05 was considered statistically significant.

## Results

### Characteristics of study participants

Baseline characteristics of participants by cases and controls are shown in Table 1. There was no significant difference between the two groups in smoking, drinking, physical activity and the level of LDL-c. However, compared with controls, the values of BMI, waist-height ratio, FPG, family history of diabetes and the level of TC, TG, were significant higher and the level of HDL-c were lower among cases at the beginning of the study.

**Table 1:** Baseline characteristics between incident cases of T2DM and control subjects

Phenotypes	Cases (*n* = 538)	Controls (n = 538)	χ^2^/Z value	*P* value
Sex (male)	195 (36.25)	195 (36.25)	0.00	Matching value
Age (years)	59.0 (51.0–66.0)	59.0 (51.0–66.0)	0.02	Matching value
BMI (kg/m2)	26.39 (23.78–28.77)	24.18 (22.02–26.57)	8.51	< 0.0001
Waist-height ratio	0.56 (0.52–0.61)	0.52 (0.48–0.57)	8.91	< 0.0001
Smoking				
Yes	132 (24.54)	142 (26.39)	0.49	0.484
No	406 (75.46)	396 (73.61)		
Drinking				
Yes	68 (12.64)	49 (9.11)	3.46	0.063
No	470 (87.36)	489 (90.89)		
Physical activity				
Low	269 (50.00)	286 (53.16)	1.15	0.562
Moderate	111 (20.63)	107 (19.89)		
High	158 (29.37)	145 (26.95)		
Family history of diabetes				
Yes	45 (10.84)	25 (5.94)	6.55	0.010
No	370 (89.16)	396 (94.06)		
FPG (mmol/L)	5.98 (5.48–6.44)	5.31 (4.97–5.66)	14.70	< 0.001
SBP (mmHg)	130.83 (117.00–145.83)	122.33 (111.92–137.33)	5.44	< 0.001
DBP (mmHg)	82.33 (74.33–91.08)	78.33 (70.67–86.33)	5.31	< 0.001
TC (mmol/L)	4.65 (4.10–5.29)	4.46 (3.89–5.07)	3.65	< 0.001
TG (mmol/L)	1.85 (1.22–2.86)	1.44 (1.03–2.05)	7.07	< 0.001
HDL-C (mmol/L)	1.09 (0.95–1.24)	1.15 (1.00–1.32)	4.56	< 0.001
LDL-C (mmol/L)	2.7 (2.20–3.10)	2.60 (2.10–3.10)	1.11	0.268

*BMI* body mass index, *FPG* fasting plasma glucose, *TC* total cholesterol, *TG* triglycerides, *HDL-C* high density lipoprotein, *LDL-C* low density lipoprotein

Data are number (percentage) or median (interquartile range)

*P* value for comparison between cases and controls

Mann-Whitney U test or χ2 test were used to test differences between groups

All SNPs for *GCKR* and *G6PC2* were in Hardy-Weinberg equilibrium (*P* > 0.05). Genotypic and allelic distributions of SNPs for *GCKR* and *G6PC2* at baseline are given in Additional file 1: Table S1. There was no difference in the distribution of SNPs of them (*P* > 0.05).

### Association of genetic variants of *GCKR* and *G6PC2* with the risk for T2DM

Table 2 reports the association between six SNPs and the risk for T2DM, along with their adjusted ORs. As subjects with rs2293572-GG genotype was uncommon, we combined the GC and GG genotypes in the analysis. Stratified Cox proportional hazards regression analysis under three different genetic models found that the C allele of rs780094 was significantly associated with an increased risk of T2DM after adjustment for BMI, BMI-change, smoking, drinking, and family history of diabetes (adjusted OR = 1.779, 95% CI 1.028–3.078, *p* = 0.040). In addition, the multivariable-adjusted ORs for each T-allele of rs780094 was 1.334 (95% CI 1.014–1.755). For SNPs of *G6PC2*, the GC genotype and dominant model (CC + GC vs. GG) of rs492594 were significantly associated with a decreased risk for T2DM (adjusted OR = 0.567, 95% CI = 0.362–0.888, *p* = 0.013; 0.601, 95% CI = 0.394–0.916, *p* = 0.018).

**Table 2:** Associations of *GCKR* and *G6PC2* SNPs with risk of T2DM

Genotype	Crude odds ratio (95% CI)	*P* value	Adjusted odds ratio (95% CI)	*P*^*a*^ value
rs780094				
TT	1.00		1.00	
CT	0.889 (0.643–1.229)	0.476	1.296 (0.811–2.072)	0.278
CC	1.058 (0.726–1.541)	0.768	1.779 (1.028–3.078)	0.040
each C-allele increase	1.028 (0.852–1.240)	0.774	1.334 (1.014–1.755)	0.039
Dominant model				
CC + CT vs. TT	0.941 (0.694–1.277)	0.697	1.434 (0.918–2.240)	0.113
Recessive model				
CC vs. CT + TT	1.145 (0.842–1.557)	0.389	1.490 (0.955–2.324)	0.079
rs2293572				
CC	1.00	–	1.00	–
GC + GG	1.177 (0.872–1.589)	0.286	0.948 (0.625–1.438)	0.803
rs1260326				
CC	1.00		1.00	
CT	0.925 (0.668–1.281)	0.639	1.334 (0.831–2.141)	0.233
TT	0.972 (0.669–1.412)	0.972	1.666 (0.963–2.880)	0.068
each T-allele increase	0.986 (0.819–1.189)	0.886	1.290 (0.981–1.696)	0.068
Dominant model				
TT + CT vs. CC	0.940 (0.692–1.279)	0.695	1.433 (0.914–2.247)	0.117
Recessive model				
TT vs. CT + CC	1.024 (0.757–1.386)	0.877	1.364 (0.881–2.111)	0.164
rs492594				
GG	1.00	–	1.00	–
GC	0.724 (0.523–1.003)	0.052	0.567 (0.362–0.888)	0.013
CC	0.832 (0.569–1.219)	0.345	0.678 (0.401–1.147)	0.147
each C-allele increase	0.907 (0.750–1.096)	0.311	0.813 (0.626–1.055)	0.119
Dominant model				
CC + GC vs. GG	0.758 (0.558–1.029)	0.076	0.601 (0.394–0.916)	0.018
Recessive model				
CC vs. GG + GC	1.026 (0.748–1.408)	0.872	0.977 (0.633–1.509)	0.916
rs16856187				
AA	1.00	–	1.00	–
CA	0.991 (0.762–1.289)	0.948	1.037 (0.729–1.476)	0.839
CC	0.929 (0.547–1.580)	0.787	1.552 (0.656–3.670)	0.317
each C-allele increase	0.978 (0.794–1.204)	0.832	1.113 (0.829–1.495)	0.475
Dominant model				
CA + CC vs. AA	0.983 (0.763–1.268)	0.897	1.077 (0.764–1.519)	0.671
Recessive model				
CC vs. AA+CA	0.933 (0.558–1.562)	0.793	1.526 (0.655–3.556)	0.327
rs13387347				
TT	1.00	–	1.00	–
CT	0.892 (0.662–1.203)	0.764	0.802 (0.533–1.206)	0.289
CC	1.080 (0.732–1.592)	0.699	1.469 (0.850–2.539)	0.169
each C-allele increase	1.014 (0.840–1.225)	0.885	1.117 (0.862–1.447)	0.401
Dominant model				
CT + CC vs. TT	0.941 (0.711–1.245)	0.668	0.947 (0.649–1.381)	0.777
Recessive model				
CC vs. TT + CT	1.153 (0.813–1.633)	0.425	1.651 (0.998–2.731)	0.051

Adjusted for BMI, BMI-change, smoking, drinking, family history of diabetes

*P*^***a***^ value is adjusted result

However, the other SNPs (*GCKR* rs1260326, rs2293572 and *G6PC2* rs13387347, rs16856187) were not found to be associated with T2DM (*p* > 0.05) in this population.

## Haplotype analyses

**Fig. 1:**
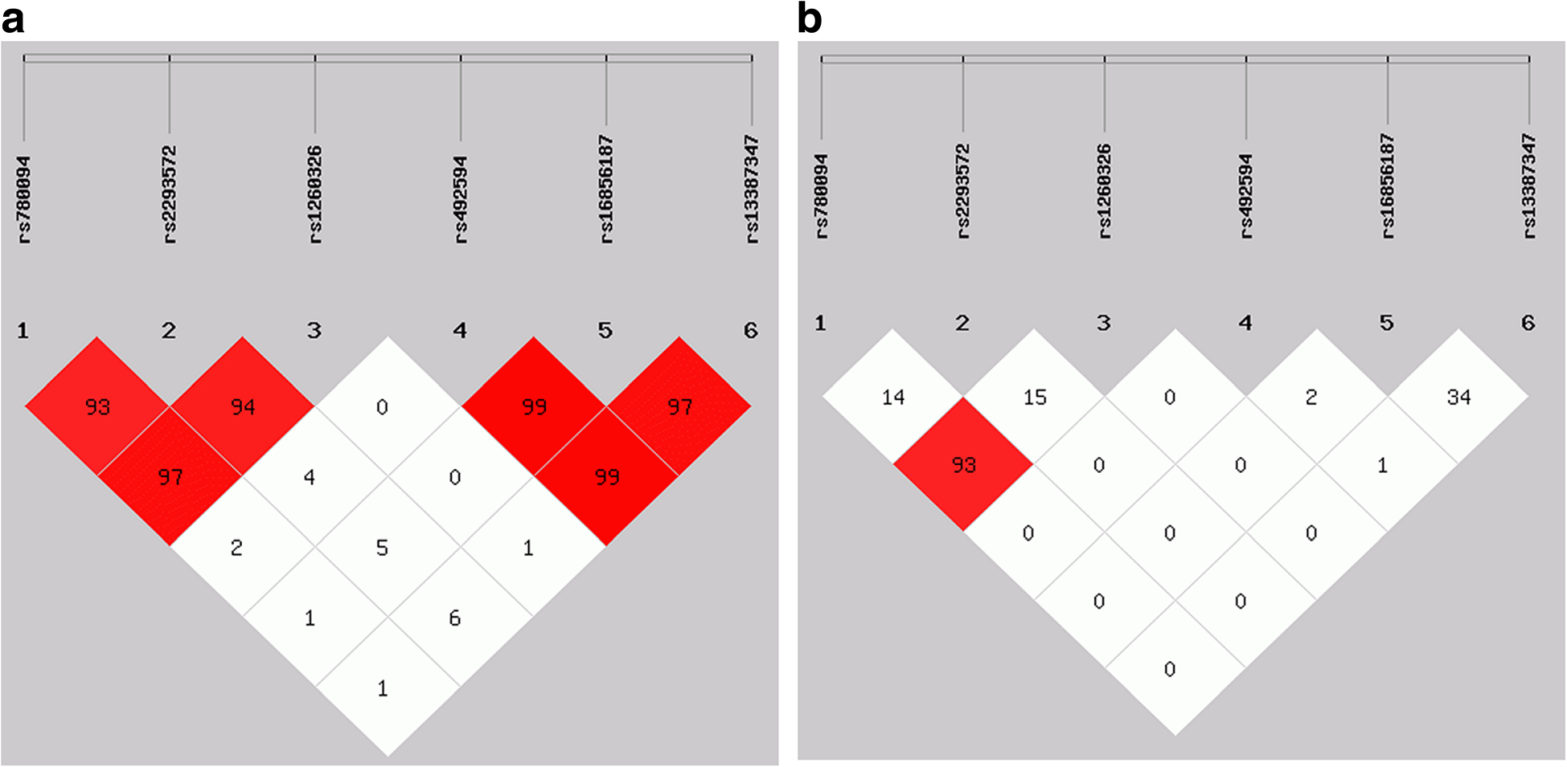
Analysis of linkage disequilibrium of the 6 SNPs. **a** The color and figure were determined by the value of D’. **b** The color and figure were determined by the value of r2. SNPs = single-nucleotide polymorphisms

The SHEsis online program was used to analyze the degree of linkage disequilibrium of the six SNPs and haplotypes in this study. The results showed that there was linkage equilibrium between the *GCKR* and *G6PC2* gene, as shown in Fig. 1. For SNPs of the *GCKR* gene, 3 haplotypes each with a frequency greater than 3% were detected due to linkage disequilibrium, and 3 haplotypes for 3 SNPs of the *G6PC2* gene were also detected. Haplotype analysis showed that there were no significant differences in frequency distribution between the 2 groups for both the *GCKR* and *G6PC2* genes (*P* > 0.05) as shown in Additional file 1: Table S2.

### SNP-SNP and haplotype-based interaction

The generalized multifactor dimensionality reduction (GMDR) v0.9 was applied in this research to detect the interaction of the 6 selected SNPs in *GCKR* and *G6PC2*. Table 3 provided the best model testing by GMDR. In all of the models the combination of *GCKR* rs2293572 and *G6PC2* rs492594 formed the best model with a statistically significant *p* value of 0.011, a maximum testing balanced accuracy of 55.0% and the biggest CV consistency (10/10) after adjusting the covariables including age, sex, smoking, drinking, BMI, BMI-change and family history of diabetes.

**Table 3:** Interaction results between the *GCKR* and *G6PC2* SNPs by GMDR

Model	Covariates	TBA^a^	CVC^b^	*P* value
rs492594	none	0.514	9/10	0.377
	adjusted	0.516	9/10	0.377
rs2293572, rs492594	none	0.544	10/10	0.055
	adjusted	0.552	10/10	0.011
rs2293572, rs492594, rs13387347	none	0.551	9/10	0.172
	adjusted	0.548	8/10	0.055

a Testing balance accuracy

b Cross-validation consistency

Adjusted covariates included sex, age, BMI, BMI-change, smoking, drinking and family history of diabetes

To obtain the ORs and 95% CIs for the interaction effect of rs2293572 and rs492594 on T2DM risk under different genetic models, we conducted SNP-SNP interaction by using unconditional logistic regression adjusted variables including covariables (age, sex, BMI, BMI-change, smoking, drinking, family history of diabetes), each with two SNPs and their interaction term. As shown in Table 4, the interaction was significant under additive-additive conditions (OR = 1.695, 95% CI 1.125–2.552; *p* = 0.012). The parameter of the partial interaction term could not be estimated in the logistic regression model because the recessive model of rs2293572 was not established. Moreover, no statistically significant interaction was found for other SNP-SNP pairs (data no shown).

**Table 4:** Single nucleotide polymorphism rs2293572- single nucleotide polymorphism rs492594 interaction under different genetic models

Genetic model	Variable	OR (95% CI)	*P*^a^ value
Additive×additive	rs2293572	0.429 (0.191–0.965)	0.041
	rs492594	0.404 (0.228–0.717)	0.002
	rs229357 × rs492594	1.695 (1.125–2.552)	0.012
Dominant×dominant	rs2293572	0.791 (0.503–1.243)	0.309
	rs492594	0.726 (0.578–0.912)	0.006
	rs229357 × rs492594	1.895 (1.036–3.466)	0.038

Adjusted for age, sex, BMI, BMI-change, smoking, drinking, family history of diabetes

*P*^***a***^ value is adjusted result

**Fig. 2:**
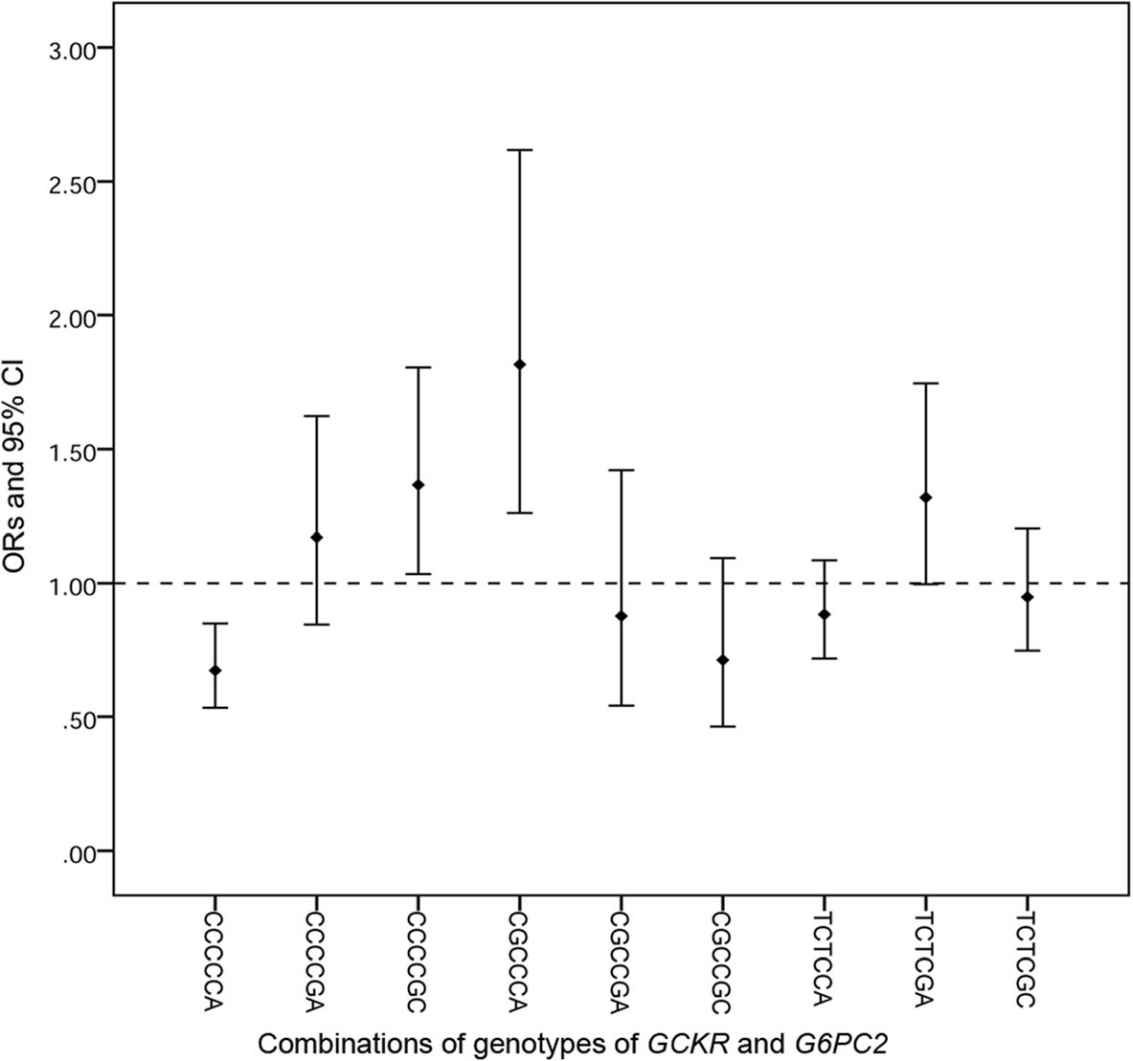
Odds-ratio (ORs) and 95% confidence intervals for T2DM estimated for the genotypic combinations of the SNPs of two genes of *GCKR* and *G6PC2*

In order to further explore the effect of gene-gene interaction, we constructed haplotypes analysis use SHEsis online. The haplotypes of these 2 genes from left to right were the alleles of rs780094, rs2293572, rs1260326, rs492594, rs16856187 and rs13387347. Ten haplotypes, each with a frequency greater than 3% were detected. Haplotype comparison analysis indicated that CCCCGC and CGCCCA haplotypes significantly increased the risk of T2DM (OR = 1.366, 95% CI 1.034–1.806, *p* = 0.028 and 1.817, 95% CI 1.261–2.618, *p* = 0.001, respectively). However, the CCCCCA haplotype correlates with lower susceptibility to T2DM (*p* = 0.000) (Additional file 1: Table S3 and Fig. 2).

## Discussion

The human *GCKR* and *G6PC2* genes are both located on chromosome 2. Glucokinase (GCK) plays a central role in the sensing of glucose in pancreatic beta-cells and parenchymal cells of liver. [17] *GCKR* can competitively inhibit GCK, which plays a major role in the regulation of insulin secretion and glycogen metabolism and is considered as a potential susceptibility gene for T2DM. [18] *G6PC2* is another important glucose related metabolism gene, principally expressed in the beta cells of pancreatic islets. [19, 20] *GCKR* and *G6PC2* encode different enzymes that may jointly regulate glucose homeostasis, effectively establishing the glucose set point. In the current study, we selected the loci of rs780094, rs2293572, rs1260326, rs492594, rs16856187 and rs13387347 from the *GCKR* and *G6PC2* genes and evaluated the association between these loci and T2DM as well as the interaction between the *GCKR* and *G6PC2* genes, which have never been reported either for the Chinese or the global population.

The results show that BMI, waist-height ratio and the levels of FPG, TC, TG and HDL-C of the T2DM group were significantly higher than those of non-T2DM at baseline group. These findings are similar to a study on Southern Han Chinese ancestry [21] In addition, the present study found that the T2DM risk was significantly higher in carriers with the C allele of rs780094 in *GCKR*. The GC genotype and dominant model of rs492594 in *G6PC2* were significantly associated with a decreased risk of T2DM. The conclusions were in line with a large scale meta-analyses by Wang et al. which indicated that *GCKR* rs780094 variants contributed to high cross-ethnicity risk for development of T2DM, with OR values (95% CI) of 1.08 (1.05–1.12) [6]. On the other hand, a study of Southern Han Chinese demonstrated that rs780094 was significantly associated with T2DM. [21] Hence, *GCKR* is thought to increase the risk of T2DM by regulating glucose levels. *G6PC2* rs16856187 was also found to be associated with T2DM in a Southern Han Chinese population [22], and rs560887 was associated with T2DM in Caucasians [23]. Nevertheless, our study did not detect a significant difference in these genotype and allele frequencies between T2DM and control groups. Besides, we also found that the rs492594 of *G6PC2* had a protective role in the pathogenesis of T2DM, nevertheless, this effect was inverted when investigating the interaction between SNP-SNP, which was not detected in other studies. These contradictory results may be affected by sample size and different types of research design. The matched nested case-control study can combine the advantages of prospective and case-control designs, effectively avoiding the potential reverse causality and confounders that are more likely to occur in cross-sectional studies, which can increase the credibility of the results.

This study is the first to identify a significant interactive effect between the rs2293572 polymorphism in *GCKR* and the rs492594 in the *G6PC2* gene on T2DM risk. It is interesting to note that although the genetic variants in the rs2293572 of *GCKR* gene did not have noticeable effects on T2DM, their interplay with genetic variants in another gene were found to have a greater effect. A carrier of the rs2293572 G allele in *GCKR* and the rs492594 C allele in *G6PC2* on average has a 1.65-fold higher risk of T2DM compared with a noncarrier under the additive model. By taking into account the epistatic interactions between potential risk loci, genetic variants, which might otherwise have remained undetected, were identified successfully here.

The role of genes in the pathogenesis of T2DM is complex. Genetic susceptibility is always inherited in the form of haplotypes, and there is more powerful statistical efficacy to identify a complex association between a pair of SNPs and a trait using haplotypes than with a univariate analysis or the interaction term. [24–26] In order to further probe the interaction effect, we conducted an analysis of haplotypes of the *GCKR* and *G6PC2* genes. Our results suggest that the interaction of the haplotypes CGC (*GCKR* gene) and GCC and CAT (*G6PC2* gene) increased the susceptibility to T2DM, while the presence of CCC (*GCKR* gene) and CAT (*G6PC2* gene) lowered the risk of T2DM. However, the mechanism of the interaction between *GCKR* and *G6PC2* genes remains unclear. There is still a need for further study at the molecular level on whether there is an association between gene regulation and expression.

The strengths of our study include its prospective study design with comprehensive evaluation of known and underlying confounding factors, blood samples, and *GCKR* and *G6PC2* genotypes in a well-characterized population. However, several limitations for this study should be considered. First, just 6 SNPs within the *GCKR* and *G6PC2* gene were chosen, and more SNPs should be included in the further studies. Second, gene-environment interaction should be investigated in future studies. Third, the results obtained in the current study should be checked in future studies with a larger sample size and in different nationalities.

## Conclusions

In conclusion, the results suggest that the C allele of rs780094 in *GCKR* was associated with an increased risk for T2DM. However, the rs492594-C allele in *G6PC2* was associated with a decreased risk of T2DM in this Han Chinese population. We also found a significant SNP-SNP interaction between rs2293572 and rs492594, and that participants with the CCCCGC and CGCCCA haplotypes had a significantly increased the risk of T2DM, while the CCCCCA haplotype conferred lower susceptibility to T2DM. The associations of gene-gene with incident of T2DM might infer potential mechanisms underlying the pathogenesis for T2DM.

## Additional file

Additional file 1: **Table S1** Genotypic and allelic distributions of single nucleotide polymorphisms (SNPs) of *GCKR* and *G6PC2*. **Table S2** Associations between the *GCKR* and *G6PC2* gene haplotypes and T2DM. **Table S3** Distribution of haplotypes of the *G6PC2* and *GCKR* genes in case and control groups. (DOCX 28 kb)

## Abbreviations

BMI: Body mass index
CI: confidence intervals
FPG: Fasting plasma glucose
G6PC2: Glucose-6-phosphatase catalytic subunit 2
GCKR: Glucokinase regulatory protein
GMDR: Generalized multifactor dimensionality reduction
HDL-C: High-density lipoprotein cholesterol
HWE: Hardy-Weinberg Equilibrium
LD: Linkage disequilibrium
LDL-C: Low-density lipoprotein cholesterol
OR: Odds ratio
SNPs: Single-nucleotide polymorphisms
T2DM: Type 2 diabetes mellitus
TC: Total cholesterol
TG: Triglycerides
