# Supplementary material for: Gene-gene interactions lead to higher risk for development of type 2 diabetes in a Chinese Han population: a prospective nested case-control study
Source: Lipids Health Dis. 2018 Jul 28;17:179. doi: 10.1186/s12944-018-0813-6 (PMC6064617; doi:10.1186/s12944-018-0813-6)
Supplement: Supplementary file 1 — Table S1 Genotypic and allelic distributions of single nucleotide polymorphisms (SNPs) of GCKR and G6PC2. Table S2 Associations between the GCKR and G6PC2 gene haplotypes and T2DM. Table S3 Distribution of haplotypes of the G6PC2 and GCKR genes in case and control groups. (DOCX 28 kb) [file 12944_2018_813_MOESM1_ESM.docx]

**Gene-gene interactions lead to higher risk for development of type 2 diabetes in a Chinese Han population: a prospective nested case-control study**

Wen Zhou^1^, Yu-qian Li^2^, Lulu Zhang^1^, Yuanyuan Shi^1^, Chongjian Wang^1^, Dongdong Zhang^1^, Xuejiao Liu^1^, Zhen-xing Mao^1^, Linlin Li^1^*

^1^*Department of Epidemiology and Health Statistics, College of Public Health, Zhengzhou University, 100 Kexue Avenue, Zhengzhou, Henan 450001, China*

^2^*Department of Clinical Pharmacology, School of Pharmaceutical Science, Zhengzhou University, 100 Kexue Avenue, Zhengzhou, Henan 450001, China*

*All authors declared no conflict of interest.*

**Correspondence author:*

Linlin Li, *100 Kexue Avenue, Zhengzhou, Henan 450001, China.*

*E-mail address:* *[lilinlin@zzu.edu.cn](mailto:lilinlin@zzu.edu.cn); Tel.: +86-13607688936; Fax: +0371-67781868*

Word count: 2854

Number of tables 4

Number of figures: 2

Number of supplementary digital content files: 3

**Table S1.** Genotypic and allelic distributions of single nucleotide polymorphisms (SNPs) of *GCKR* and G6PC2.

| SNP | Genotype/allele | Cases/controls | χ^2^ | *P* value | *P*^a^ value |
| --- | --- | --- | --- | --- | --- |
| rs780094 | TT | 137/134 | 0.41 | 0.816 | 0.260 |
|  | CT | 272/282 |  |  |  |
|  | CC | 129/122 |  |  |  |
|  | T | 546/550 | 0.03 | 0.863 |  |
|  | C | 530/526 |  |  |  |
| rs2293572 | CC | 382/403 | 2.70 | 0.259 | 0.574 |
|  | GC | 143/127 |  |  |  |
|  | GG | 13/8 |  |  |  |
|  | C | 907/933 | 2.53 | 0.111 |  |
|  | G | 169/143 |  |  |  |
| rs1260326 | CC | 131/131 | 0.14 | 0.932 | 0.730 |
|  | CT | 278/273 |  |  |  |
|  | TT | 129/134 |  |  |  |
|  | C | 540/535 | 0.05 | 0.829 |  |
|  | T | 536/541 |  |  |  |
| rs492594 | GG | 178/146 | 4.54 | 0.103 | 0.498 |
|  | GC | 255/276 |  |  |  |
|  | CC | 105/116 |  |  |  |
|  | G | 611/568 | 3.47 | 0.063 |  |
|  | C | 465/508 |  |  |  |
| rs16856187 | AA | 252/254 | 0.433 | 0.805 | 0.173 |
|  | CA | 237/241 |  |  |  |
|  | CC | 49/43 |  |  |  |
|  | A | 741/749 | 0.14 | 0.709 |  |
|  | C | 335/327 |  |  |  |
| rs13387347 | TT | 153/156 | 1.751 | 0.417 | 0.093 |
|  | CT | 271/285 |  |  |  |
|  | CC | 114/97 |  |  |  |
|  | T | 577/597 | 0.75 | 0.387 |  |
|  | C | 499/479 |  |  |  |

*P*^a^ value is the result from Hardy-Weinberg equilibrium.

**Table S2.** Associations between the *GCKR* and *G6PC2*gene haplotypes and T2DM.

|  | Haplotype | Cases/controls (n) | Frequencies (%) | *P* value | OR (95% CI) |
| --- | --- | --- | --- | --- | --- |
| Block 1 | CCC | 370/390 | 34.4/36.2 | 0.374 | 0.923 (0.772-1.102) |
|  | CGC | 163/140 | 15.1/13.0 | 0.166 | 1.188 (0.931-1.516) |
|  | TCT | 521/523 | 48.4/48.6 | 0.905 | 0.990 (0.834-1.174) |
| Block 2 | CCA | 463/502 | 43.0/46.6 | 0.081 | 0.859 (0.725-1.019) |
|  | CGA | 246/214 | 22.8/19.9 | 0.110 | 1.183(0.962-1.455) |
|  | CGC | 333/321 | 30.9/29.8 | 0.604 | 1.050 (0.873-1.262) |
|  | TGA | 32/33 | 3.0/3.1 | 0.873 | 0.960 (0.586-1.575) |

Block 1 include rs780094, rs2293572 and rs1260326; Block 2 include rs492594, rs16856187 and rs13387347.

Cases/controls and frequencies were estimated by SHEsis online.

OR, odds ratio; CI, confidence interval.

**Table S3.** Distribution of haplotypes of the *G6PC2* and *GCKR* genes in case and control groups.

| Haplotype | Cases |  |  | Controls |  | χ^2^ | *P* value | OR (95% CI) |
| --- | --- | --- | --- | --- | --- | --- | --- | --- |
|  | N= 1086 | % |  | N=1082 | % |  |  |  |
| CCCCCA | 150 | 13.9 |  | 206 | 19.2 | 11.32 | 0.000 | 0.673 (0.534-0.849) |
| CCCCGA | 84 | 7.8 |  | 72 | 6.7 | 0.90 | 0.343 | 1.171 (0.845-1.624) |
| CCCCGC | 129 | 1.2 |  | 97 | 9.0 | 4.84 | 0.028 | 1.366 (1.034-1.806) |
| CGCCCA | 84 | 7.8 |  | 48 | 4.5 | 10.51 | 0.001 | 1.817 (1.261-2.618) |
| CGCCGA | 32 | 3.0 |  | 36 | 3.4 | 0.29 | 0.593 | 0.877 (0.541-1.421) |
| CGCCGC | 37 | 3.5 |  | 52 | 4.8 | 2.43 | 0.119 | 0.712 (0.463-1.094) |
| TCTCCA | 222 | 20.6 |  | 243 | 22.5 | 1.39 | 0.239 | 0.883 (0.718-1.086) |
| TCTCGA | 125 | 11.6 |  | 97 | 9.0 | 3.751 | 0.053 | 1.319 (0.996-1.746) |
| TCTCGC | 157 | 14.6 |  | 163 | 15.2 | 0.194 | 0.660 | 0.948 (0.747-1.203) |

Cases/controls and frequencies were estimated by SHEsis online.

OR, odds ratio; CI, confidence interval.
